# Paine on Treating Gold Fillings, &c

**Published:** 1857-09

**Authors:** G. L. Paine


### TREATMENT OF GOLD FILINGS, ETC.

BY G. L. PAINE.

Having passed the magnet through the filings, to remove therefrom the iron and steel, put them in a glass vessel—a tumbler for instance—and pour on them nitric acid. The tumbler may be set in a vessel of water, which should be gradually raised toward the boiling point, in order to accelerate the action of the nitric acid. This action should be continued for several hours, to insure the destruction of such base metals as may be mixed with the gold filings. Pour off the acid, and wash in warm water till the filings are perfectly clean; when dry, melt in a clean crucible with saleratus, then with borax, and in a majority of cases, the resulting button of gold will work as well as the best gold coin.

The metals which we use for male casts, when poured into the mould, will often bubble, or boil, and thus damage the cast. This may be prevented by piercing the bottom of the mould with a number of fine holes, made with a darning needle, or a fine, smooth, straight wire. These little apertures afford an outlet for the steam generated by the hot metal coming in contact with the moist sand, and the integrity of the cast is thus secured.

The warping or springing of a full upper set may to a very great extent be prevented by attaching to the plate just behind the heel teeth a couple of points or studs, one on each side. Between these studs pass a steel bar or iron wire, so stiff that it will not be affected by the heat required to solder the case, and the result will be that, if care be taken in heating up the case gradually and evenly, the plate will have sprung but little, if any.

